# Supplementary material for: Development of a computer-aided design software for the quantitative evaluation of aesthetic damage
Source: PLoS One. 2019 Dec 18;14(12):e0226322. doi: 10.1371/journal.pone.0226322 (PMC6919621; doi:10.1371/journal.pone.0226322)
Supplement: S1 Code site — 1. (ZIP) [file pone.0226322.s001.zip › [EESC_jr][Projeto]Relatorio_07_21/reports/templates_zip/gentelella-master/vendors/Flot/examples/image/index.html]

Flot Examples: Image Plots


## Image Plots

The Cat's Eye Nebula (picture from Hubble).

With the image plugin, you can plot static images against a set of axes. This is for useful for adding ticks to complex prerendered visualizations. Instead of inputting data points, you specify the images and where their two opposite corners are supposed to be in plot space.

Images represent a little further complication because you need to make sure they are loaded before you can use them (Flot skips incomplete images). The plugin comes with a couple of helpers for doing that.

Copyright © 2007 - 2014 IOLA and Ole Laursen
